# Primary Tuberculous Mastitis: The first report from Syria

**DOI:** 10.1016/j.ijscr.2020.02.007

**Published:** 2020-02-06

**Authors:** Sadallah Kayali, Aos Alhamid, Ayman Kayali, Aghyad Kudra Danial, Muhamad Zakaria Brimo Alsaman, Ahmad Alhamid, Kusay Ayoub

**Affiliations:** a Faculty of Medicine, University of Aleppo, Aleppo, Syria; b Department of Surgery, Aleppo University Hospital, Aleppo, Syria; c General Surgery Department, Aleppo University Hospital, Aleppo, Syria

**Keywords:** Primary breast tuberculosis, Case report

## Abstract

**Introduction:**

Primary breast tuberculosis is a rare form of extrapulmonary tuberculosis even in endemic regions. To our knowledge, this is the first report of Primary breast tuberculosis from Syria.

**Case presentation:**

We report a case of a 37-year-old female who admitted to the surgical clinic with a 4-month history of gradually growing mass in the breast. On physical examination there were a palpable mass, painful superficial abscess in her left lateral upper quarter of breast, redness and nipple retraction and ulceration. The patient history and physical examination were clear except for uncontrolled hypothyroidism. Radiological tests including mammography, echography and laboratory investigations were performed. The patient underwent lumpectomy. Histopathologic examination showed caseating Tuberculous Mastitis and a large tuberculous abscess, with no malignancy. Patient was put on anti-tubercular chemotherapy, but recurred after three months with three masses in the same area because she did not adhere to the treatment. Lumpectomy and Anti-tuberculous therapy were repeated again with close follow-up, and the patient recovered.

**Discussion:**

Primary breast tuberculosis forms about 0.025-0.1 % of all surgically treated breast diseases. Diagnosis is based on bacteriological and histological examination. We can get higher accuracy in diagnosis by biopsy such as a core needle or surgical biopsy, surgical biopsy is necessary to confirm the diagnosis of tuberculous mastitis.

**Conclusion:**

Tuberculous mastitis is extremely rare variant of extrapulmonary tuberculosis. However, it should be kept in the mind of physicians and pathologists while approaching a breast mass, especially in endemic area.

## Highlights

• Breast TB is not commonly seen in medical practice.
• Surgical biopsy is necessary to confirm the diagnosis of tuberculous mastitis.
• Usually Anti-tuberculous therapy is the main treatment for breast TB.
• Minimal surgery is performed to remove any residual lesions.

## 1 Introduction

Tuberculosis (TB) is one of the most widespread infections in the world. In 2018, WHO reported ten million new TB cases, the vast majority of them are in developing countries [1]. However, breast TB (tuberculous mastitis) is not commonly seen in medical practice. It was first described by Sir Astley Cooper in 1829 [2]. The incidence of Tuberculous mastitis ranges from 0.1 % in developed countries to about 4 % in highly endemic countries [3,4]. Tuberculous mastitis is usually diagnosed in young lactating multiparous women [5,6].

The term ‘primary tuberculous mastitis’ is specifically used for those rare cases in which tubercle bacilli infects the breast at first. ‘Secondary tuberculous mastitis’ is used when there is a tuberculous co-infection elsewhere in the body [7].

Following SCARE reporting guidelines [8], we report a rare case of primary tuberculous mastitis in a 37-year-old Syrian female, which was first suspected as a malignancy, but was then truly diagnosed after histopathologic examination of the excisional biopsy. We also discuss the treatment of the case before and after recurrence.

## 2 Case presentation

**Fig. 1:**
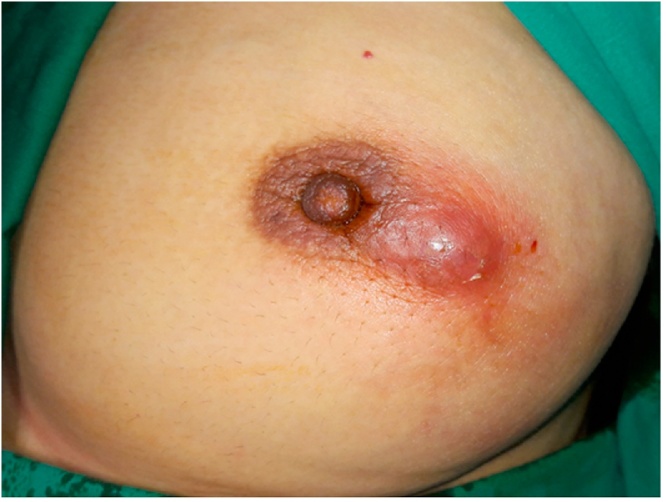
A palpable mass in the patient’s left breast, with superficial abscess and retraction and ulceration of the skin.

In April 2018, a 37-year-old Syrian female came to the surgical outclinic of our institute in Aleppo-Syria, complaining of a palpable mass in her left breast, with retraction and ulceration of the skin, pain and redness. The mass grew gradually over the period of four months (Fig. 1).

The patient is an illiterate housewife that lives in a rural area. She has one child that she has breastfed him.

The patient reported hypothyroidism. She was on l-thyroxin 50 μg/day but stopped the medicine two months ago without medical consultation. Otherwise, her past medical, surgical and medicinal history is clear, including pulmonary diseases.

Review and examination of other systems was not significant. There was no history of weight loss, loss of appetite, night sweats or fever.

The patient performed an ultrasonography (US) a month earlier. It showed a (4 × 6) cm poorly identified structure, which infiltrates in the glandular tissue and distorted the normal structure of glandular lobes, but without observing an isolated mass. No dilation in the mammary ducts was observed. The US also revealed an enlarged left axillary lymph node, 2 cm in diameter. The right breast and the right axillary region showed no abnormalities. At that time, two biopsies from the mass were performed by an interventional radiologist to investigate the mass. Histopathologic examination revealed no malignancy with acute non-specific mastitis and foci of fat necrosis.

**Fig. 2:**
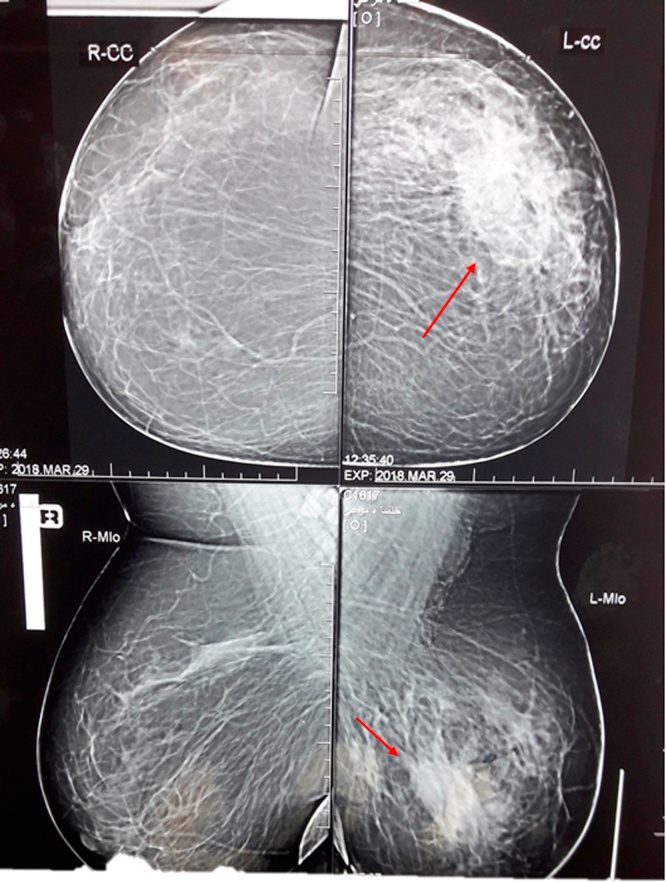
Mammography shows irregular mass (4 × 3.5 cm) in the lateral upper quadrant of the left breast.

We performed another US and mammography for the two breasts. The mammography showed two dense and disproportionate breasts, and a (4 × 3.5) cm irregular mass located in the lateral upper quadrant of the left breast (Fig. 2). For ultrasonography, the breasts were studied with a 7.5 MHz probe. We found a small, irregular, hypoechoic mass (4.5 × 3.6) cm located at 1 o’clock in the left breast, and an axillary lymph node in the left side was observed. We suspected malignancy.

Chest X-ray was normal.

The patient was admitted to our surgical department to be prepared for excisional biopsy of the mass.

Laboratory values were as follows: Hb: 10.8 mg/dl– WBC: 10800/mm^3^ - TSH: 31.4 μIU/mL- CRP : 31 mg/L - ESR : 1^st^ h 46.

Hypothyroidism was corrected before surgery by l-thyroxin.

**Fig. 3:**
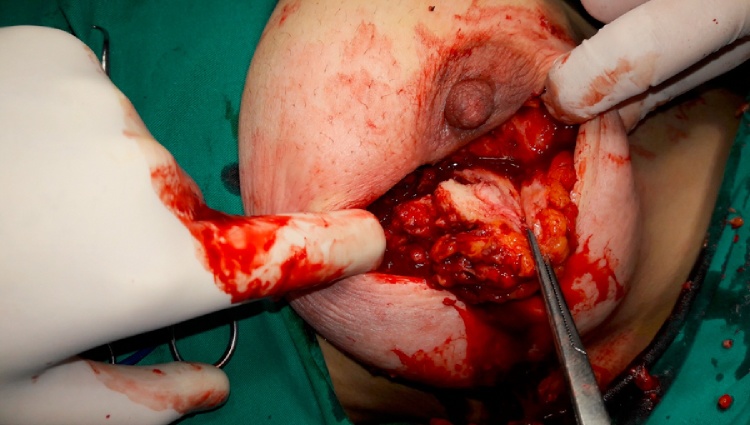
A view of the caseous granuloma during surgery.

Echography of the thyroid gland was within normal limits with some degree of atrophy. Once the patient reached euthyroidism (TSH: 2.1 μIU/mL), we performed lumpectomy under general anesthesia (Fig. 3).

The resected mass was tan and rubbery, measuring 13 ×8 ×4 cm. The cut section revealed irregular surface admixed with fat, and many dilated ducts with cystic formations and large areas of necrosis.

**Fig. 4:**
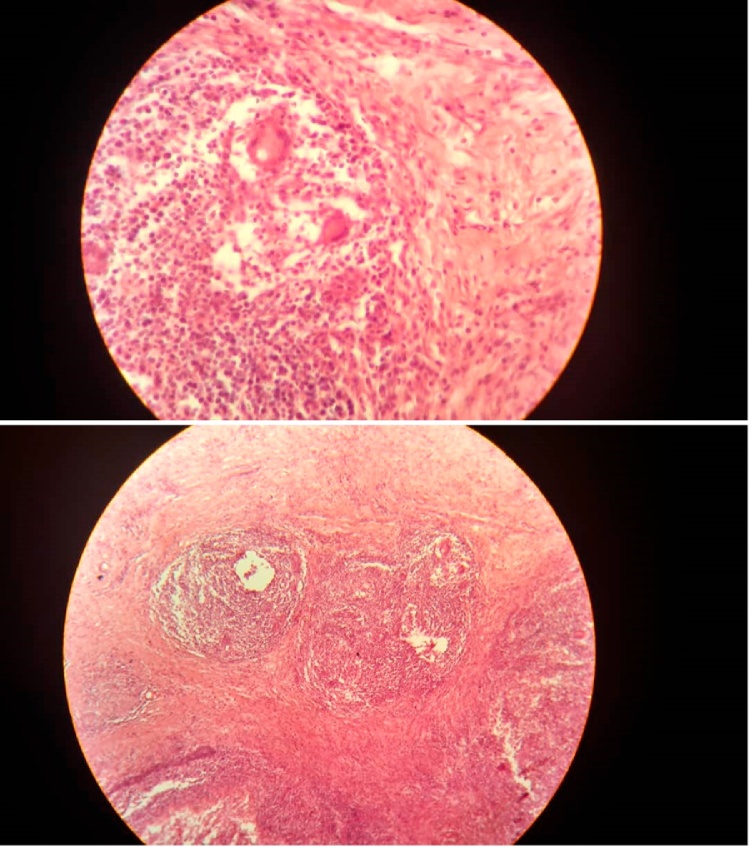
Granulomatous caseating nodules consisting of central zones of caseous necrosis, surrounded by a mixture of small lymphocytes, plasma cells, epithelioid cells and Langerhans cells.

Microscopic examination revealed granulomatous caseating tuberculous mastitis in the left breast. (Fig. 4).

Then the patient was put on (Rifampicinb150 mg, Isoniazid 75 mg, Pyrazinamide 400 mg, Ethamputol 275 mg) and after one month of treatment by ATT drugs:

HB: 14.5 mg/dl - WBC : 7500/mm^3^ -ESR : 34 1^st^ hour - CRP : 6.8 mg/L - Iron : 70 mcg/dL.

The patient did not adhere to treatment because she lives in a remote village, with limited accessibility to TB treatment facilities due to war. She came after three months with 3 masses in her left breast, The patient was sent to a radiologist and the echography revealed the presence of three masses: the first mass was (2 × 1.5) cm above the middle of the surgical incision by (2) cm with irregular borders, the second was (2 × 0.6) cm with irregular borders below the end of the lateral line of surgical incision by (2) cm, and the third was (3) mm lateral to the latter described mass.

Near the place of pervious surgical incision, there was a hypoechoic tissue (2 × 2) cm and the Doppler showed a relative increase in blood supply. The rest of glandular tissue looked normal. Three axillary lymph nodes were seen (17–13–12) mm with a normal adipose hilum.

We performed another lumpectomy. Biopsies were sent to the pathology laboratory and the result was caseating tuberculosis mastitis with abscess and fibrosis of left breast with no malignancy.

The patient was successfully treated after the excision of the three masses and returning to the ATT drugs (Rifampicin150 mg, Isoniazid 75 mg, Pyrazinamide 400 mg, Ethamputol 275 mg) for 9 months with thorough follow-up. Another echography was performed after a year and was normal and the patient recovered.

## 3 Discussion

Primary form of breast tuberculosis is a very rare type of TB [9]. It occurs in women in reproductive age groups, but it can affect the female breast at any age [12]. Breast tuberculosis can rarely affect males [10]. Tuberculous Mastitis forms about 0.025-0.1 % of all surgically treated breast diseases [11]. Breast tuberculosis is more common in developing countries and has a lower incidence in western countries [9]. Breast tissue is often not a suitable environment for tubercle bacilli to grow [13].

Risk factors include: multiparity, lactation, trauma, immunosuppression, and past history of suppurative mastitis [14].

It may present with an irregular lump in the breast, sometimes fixed to the overlying skin or the underlying muscle, and is often misdiagnosed as carcinoma or pyogenic abscess [12]. Most cases are accompanied with purulent nipple discharge, but fistulas and sinus tracts occur in advanced stages [15]. Our patient had an irregular lump without purulent nipple discharge, but with a superficial abscess.

Mammary tuberculosis may be primary or secondary. The primary breast tuberculosis is less common than secondary and occurs through skin abrasions or through the duct openings on the nipple [9]. Secondary breast tuberculosis occurs by transmission from another tuberculous focus, such as tuberculosis of the lung, ribs and lymph nodes [14]. By physical and radiological examination, we did not find any focus of tuberculosis outside the breast 50–75 % of patients have involvement of axillary nodes at the time of presentation, exactly like our patient [14].

McKeown and Wilkinson classified breast tuberculosis into five different types: Nodular tuberculous mastitis, Disseminated tuberculous mastitis, Sclerosing tuberculous mastitis, tuberculous mastitis obliterans and acute military tuberculous mastitis. The nodulocaseous form presents as a painless, slowly growing and well circumscribed mass that progresses to involve the overlying skin and may ulcerate forming discharging sinuses. The disseminated form starts with multiple foci throughout the breast that caseates later, causing sinus formation with or without painful ulceration. The sclerosing form occurs in the elderly, dominant feature being excessive fibrosis rather than caseation. It suspects with scirrhous carcinoma. Tuberculous mastitis obliterans is characterized by duct infection causing proliferation of lining epithelium with marked epithelial and periductal fibrosis. Acute miliary tuberculous mastitis is considered as a part of a generalized miliary tuberculosis [16]. Our case is of the nodulocaseous form.

There are several histopathological differential diagnoses of breast tuberculosis like fungal infection, actinomycosis, histoplasmosis, brucellosis, primary inflammatory diseases such as sarcoidosis and Wegener’s granulomatosis, plasma cell mastitis and traumatic fat necrosis [17]. It is difficult to differentiate mammary tuberculosis from carcinoma radiological patterns [9]. The diagnosis rests on bacteriological and histological examination [18]. We can get higher diagnostic accuracy by histopathologic examination of a biopsy such as a core needle or surgical biopsy [13]. Surgical biopsy is necessary to confirm the diagnosis of tuberculous mastitis, and it is a therapeutic intervention as well [12]. Detection of acid-fast bacilli (AFB) in the lesions is usually difficult. In tuberculous mastitis, AFB are identified only in 12 % of patients. However, it is usually sufficient to find the presence of caseating granulomas with Langerhans giant cells in the breast tissue, with involvement of lymph nodes. In tuberculosis-endemic countries, presence of granuloma FNAC allows experimental treatment for tuberculosis even in the absence of positive AFB and without culture results [19].

There are no specific guidelines available to treat breast tuberculosis. The disease should be treated as any other form of extrapulmonary TB. Usually Anti-tuberculous therapy is the main treatment for breast TB, and minimal surgery is performed to remove any residual lesions. Anti-tuberculous therapy comprises rifampicin, isoniazid, pyrazinamide and ethambutol [5]. Healing is usual, although often delayed. Mastectomy should be restricted to patients with persistent residual infection [18]. Our patient recurred with three masses after quitting the first treatment program. After that, the patient underwent another lumpectomy and we gave her Anti-tuberculous therapy with close follow up.

To our knowledge, this is the first report of primary tuberculous mastitis in Syria.

## 4 Conclusion

Tuberculous mastitis is extremely rare variant of extrapulmonary tuberculosis. However, it should be kept in the mind of physicians and pathologists while approaching a breast mass, especially in endemic areas. Breast TB may be misdiagnosed as a suspected malignancy. So, histopathologic examination of the excisional biopsy is the only confirmatory diagnostic test. Modern ATT, surgery and close follow-up are the curable treatment. Finally, Accessibility to TB treatment facilities should be available for all patients to avoid recurrence.

## Sources of funding

There are no sources of funding.

## Ethical approval

Not required for case reports at our hospital. Single case reports are exempt from ethical approval in our institution.

## Consent

Written informed consent was obtained from the patient for publication of this case report and accompanying images. A copy of the written consent is available for review by the Editor-in-Chief of this journal on request.

## Author contribution

Sadallah Kayali: managed the patient and did the surgery, the Supervisor, patient care.

Aos Alhamid: data collection, data interpretation and analysis, wrote the manuscript.

Ayman Kayali: data collection, data analysis, revision, corresponding author.

Muhamad Zakaria Brimo Alsaman: design of the study, revising critically, wrote the manuscript, and analysis.

Aghyad Kudra Danial: patient care, revision.

Kusay Ayoub: revising critically, patient care.

Ahmad Alhamid: data interpretation and analysis, revision.

All authors read and approved the final manuscript.

## Registration of research studies

N/A.

## Guarantor

Sadallah Kayali.

## Provenance and peer review

Not commissioned, externally peer-reviewed.

## Declaration of Competing Interest

The authors declare that they have no conflict of interest.

## References

[1] World Health Organization (2019). Global Tuberculosis Report 2019.

[2] (1829). Illustrations of the Diseases of the Breast.

[3] De Sousa R., Patil R. (2011). Breast tuberculosis or granulomatous mastitis: a diagnostic dilemma. Ann. Trop. Med. Public Health.

[4] Gon S. (2013). Tubercular Mastitis – A Great Masquerader/Tüberküloz Mastiti-Büyük Taklitçi. Turk. J. Pathol..

[5] Kao P.T., Tu M.Y., Tang S.H., Ma H.K. (2010). Tuberculosis of the breast with erythema nodosum: a case report. J. Med. Case Rep..

[6] Shinde S.R., Chandawarkar R.Y., Deshmukh S.P. (1995). Tuberculosis of the breast masquerading as carcinoma: a study of 100 patients. World J. Surg..

[7] Schaefer G. (1955). Tuberculosis of the breast: a review with the additional presentation of ten cases. Am. Rev. Tuberc. Pulmonary Dis..

[8] Agha R.A., Borrelli M.R., Farwana R., Koshy K., Fowler A., Orgill D.P., For the SCARE Group (2018). The SCARE 2018 statement: updating consensus Surgical CAse REport (SCARE) guidelines. Int. J. Surg..

[9] Madhusudhan Ks G. (2008). Primary breast tuberculosis masquerading as carcinoma. Singapore Med. J..

[10] Jaideep C., Kumar M., Khanna A.K. (1997). Male breast tuberculosis. Postgrad. Med. J..

[11] Kalarç N., Ozkan B., Bayiz H., Dursun A.B., Demirağ F. (2002). Breast tuberculosis. Breast.

[12] Rosen P.P. (2015). Specific infections. Rosen’s Breast Pathology.

[13] Luh S.P., Hsu J.D., Lai Y.S., Chen S.W. (2007). Primary tuberculous infection of breast: experiences of surgical resection for aged patients and review of literature. J. Zhejiang Univ. Sci. B.

[14] Gupta P.P., Gupta K.B., Yadav R.K., Agarwal D. (2003). Tuberculous mastitis: a review of seven consecutive cases. Indian J. Tuberc..

[15] Bhosale A.A., Joshi A.R., Ashturkar A.V., Pathak G.S. (2012). Primary tuberculosis of breast: a case series. Ann. Trop. Med. Public Health.

[16] Mckeown K.C., Wilkinson K.W. (1952). Tuberculous disease of the breast. Br. J. Surg..

[17] Thimmappa D., Mallikarjuna M.N., Vijayakumar A. (2015). Breast tuberculosis. Indian J. Surg..

[18] Bailey H., Love R.J.M.N., Mann C.V., Russell R.C.G. (2018). Bailey and Love’s Short Practice of Surgery.

[19] Tauro L.F., Martis J.S., George C., Kamath A., Lobo G., Hegde B.R. (2011). Tuberculous mastitis presenting as breast abscess. Oman Med. J..

